# Revised understanding of iatrogenic lumbosacral nerve bowstringing disease: a case report and literature review

**DOI:** 10.3389/fsurg.2025.1681708

**Published:** 2025-10-14

**Authors:** Haoran Gao, Lei Tian, Yunyan Tong, Haibo Zhang, Heling Zhang

**Affiliations:** 1 Department of Orthopedics, Sixian Hospital of Traditional Chinese Medicine, Suzhou, Anhui, China; 2 Department of Spinal Surgery, The Affiliated Hospital of Xuzhou Medical University, Xuzhou, Jiangsu, China

**Keywords:** iatrogenic lumbosacral nerve bowstringing disease, axial nerve tension, lumbarfusion with internal fixation surgery, nerve damage, intervertebral space height

## Abstract

**Background:**

Iatrogenic lumbosacral nerve bowstringing disease (ILNBD) is a severe complication of spinal surgery. The clinical presentation is often insidious and may be misdiagnosed as nerve root compression. Heightened diagnostic vigilance supported by characteristic radiological findings is essential.

**Case presentation:**

This case report a 63-year-old woman who underwent L3–L5 interbody fusion and internal fixation surgery for vertebral slippage combined with lumbar spinal stenosis. Her symptoms improved significantly on the second day after surgery, but she suddenly developed lower limb neurological dysfunction on the fifth day after surgery. Imaging studies showed that the internal fixation and interbody fusion devices were well positioned, with increased spinal canal volume and no space-occupying lesions. However, axial MRI revealed high tension of the cauda equina and positive nerve descent signs, consistent with the characteristics of bowstring disease.

**Conclusion:**

Excessive expansion of the intervertebral space can lead to sustained static traction on the nerve roots. The appropriate fusion device height should be determined through a stepwise expansion test to avoid increased axial tension on the nerves. Additionally, technical operational details can independently induce bowstring syndrome, and stepwise tension assessment is crucial for preventing traction-related nerve damage.

## Background

Iatrogenic lumbosacral nerve bowstringing disease (ILNBD) is a rare but severe complication following spinal surgery (1). Its core pathology involves axial traction injury to the dura mater or nerve roots due to surgical interventions (Iaminectomy, fusion device implantation, and multilevel correction). This results in loss of physiological buffering capacity, generating a nerve axis hypertension state analogous to a taut bowstring. Consequently, pathological traction during spinal motion is exacerbated, ultimately causing neurological dysfunction. Clinical manifestations mimic persistent nerve root compression or spinal cord lesions, including refractory pain, sensory deficits, hyperreflexia, and in severe cases, foot drop or cauda equina syndrome, significantly impairing patients’ quality of life (2, 3). This report presents a case of intractable low back pain with radiating lower extremity pain, hyperreflexia, and sensory abnormalities following lumbar fusion with internal fixation. Postoperative imaging revealed no compressive lesions within the spinal canal, but MRI indicated cauda equina hypertension (4, 5). Based on clinical features and literature review, iatrogenic traction neuropathy was diagnosed. We herein analyze the clinical characteristics, key imaging findings, and pathogenesis to enhance awareness of this subtle postoperative complication.

## Case presentation

**Figure 1:**
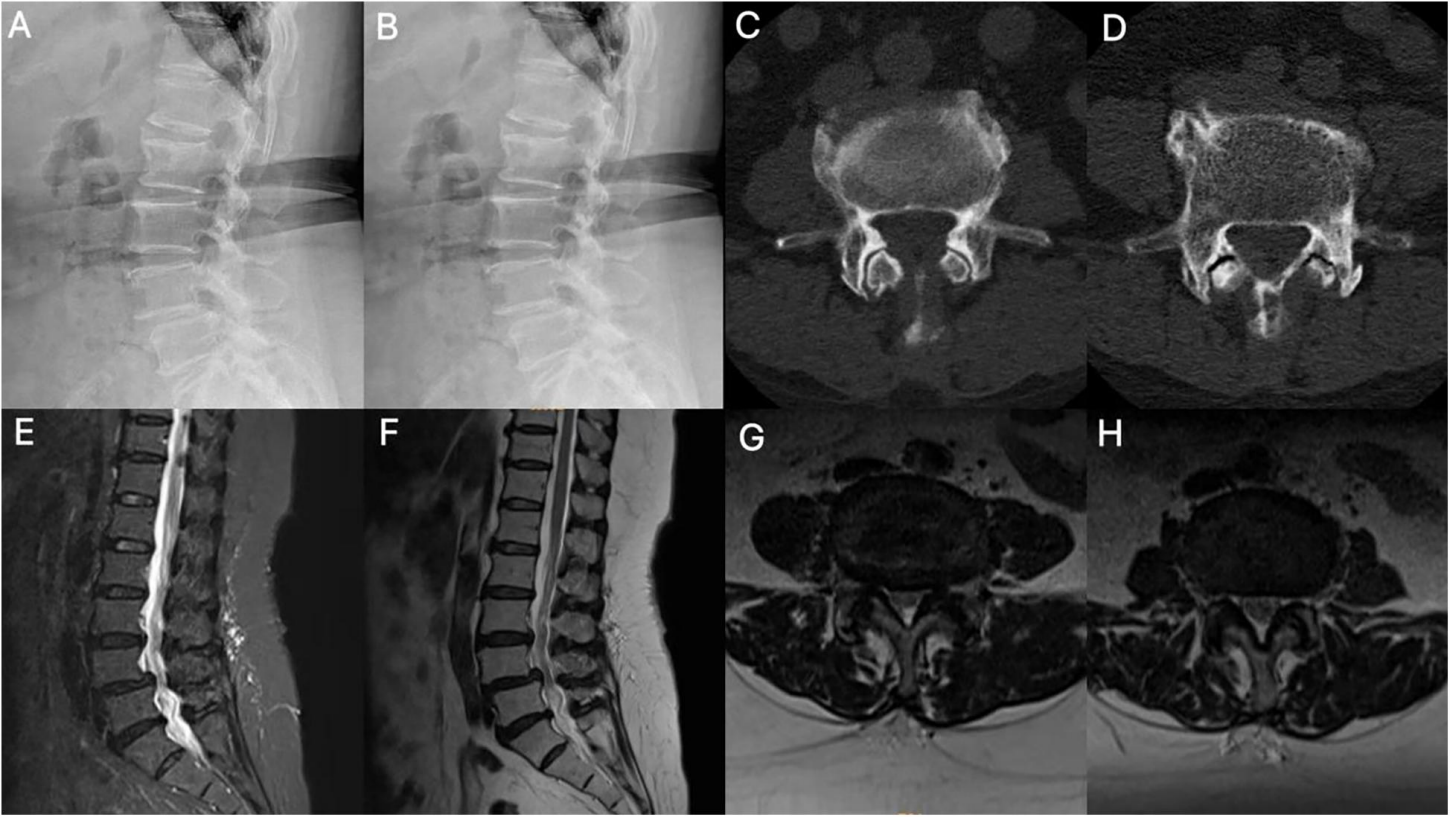
Imaging findings. **(A,B)** Dynamic lumbar radiographs demonstrate instability with spondylolisthesis. **(C,D)** Axial CT of the lumbar spine showed segments L3–4 and L4–5 with degenerative changes and lumbar disc herniation. **(E-H)** MRI shows disc herniation, folded ligamentum flavum causing pincer-type compression of the cauda equina, facet joint hypertrophy, and spinal canal stenosis.

A 63 year old female patient came to our hospital for treatment due to bilateral lower limb pain accompanied by intermittent claudication for over six months. She was diagnosed with L4 vertebral body slippage, L3–S1 lumbar disc herniation, and lumbar spinal stenosis. Her preoperative Visual Analogue Scale (VAS) pain score was 6 (Figure 1). After successful anesthesia, a midline incision is made around the L4 spinous process, exposing the L3–L5 vertebral plates, facet joints, and needle insertion points layer by layer. After confirming the gap, implant 65 * 4.5 mm universal pedicle screws, pre-bend the connecting rod and install it, appropriately widen each gap, and lock it. Bite off the spinous process of the lumbar spine, decompress the left vertebral plate, and bite the bitten vertebral plate and spinous process bone into granules for bone grafting. During the operation, spinal stenosis was observed, and decompression was performed. The compressed dura mater was restored, and the slipped vertebral body was reduced. The nerve root was protected by a rectangular incision of the posterior longitudinal ligament and annulus fibrosus. The nucleus pulposus was scraped off, the intervertebral space was treated, and the intervertebral cartilage plate was scraped off. An appropriate amount of autogenous bone was taken and placed in the intervertebral space. A 12 * 22 mm intervertebral fusion cage was placed in the L4–5 space, with the posterior edge of the cage about 5 mm away from the posterior edge of the vertebral body. The L4–5 space was appropriately compressed. Reprocess the L3–4 intervertebral space, protect the nerve root by rectangular incision of the posterior longitudinal ligament and annulus fibrosus, scrape off the nucleus pulposus, prepare the intervertebral space, scrape off the intervertebral cartilage plate, take an appropriate amount of autogenous bone and insert it into the intervertebral space, and insert a 12 * 22 mm intervertebral fusion device into the L3–4 space. Rinse the wound thoroughly, stop bleeding, and fix firmly. An x-ray shows that the pedicle screws are well positioned, the fusion device is in good position, and the slipped vertebral body has been reduced. After checking the dressing and instruments, suture layer by layer, insert a drainage tube into the incision, connect it to a negative pressure ball, close the incision, and cover it with a sterile dressing. The surgery went smoothly, with intraoperative bleeding of approximately 250 ml. The anesthesia was satisfactory, and the patient returned safely to the ward.

Reviewing the surgical procedure, it is noteworthy that before implanting the fusion device, the method for expanding the intervertebral space relied on the surgeon's tactile experience with the flexibility of the space during surgery. No intervertebral spreader was used to perform objective, stepwise testing, nor was annular tension established as a quantifiable endpoint. This approach may introduce subjective error in the final determination of intervertebral space height, potentially increasing the risk of excessive expansion. Additionally, fusion cages of identical height were implanted in the L3–4 level. During multi-level fusion procedures, particular attention must be paid to restoring intervertebral space height while avoiding excessive expansion. Such practices may reduce the redundant volume within the spinal canal and neural foramina, resulting in persistent axial traction on the cauda equina and nerve root tails. This creates a potential risk for postoperative deterioration of neurological function.

**Figure 2:**
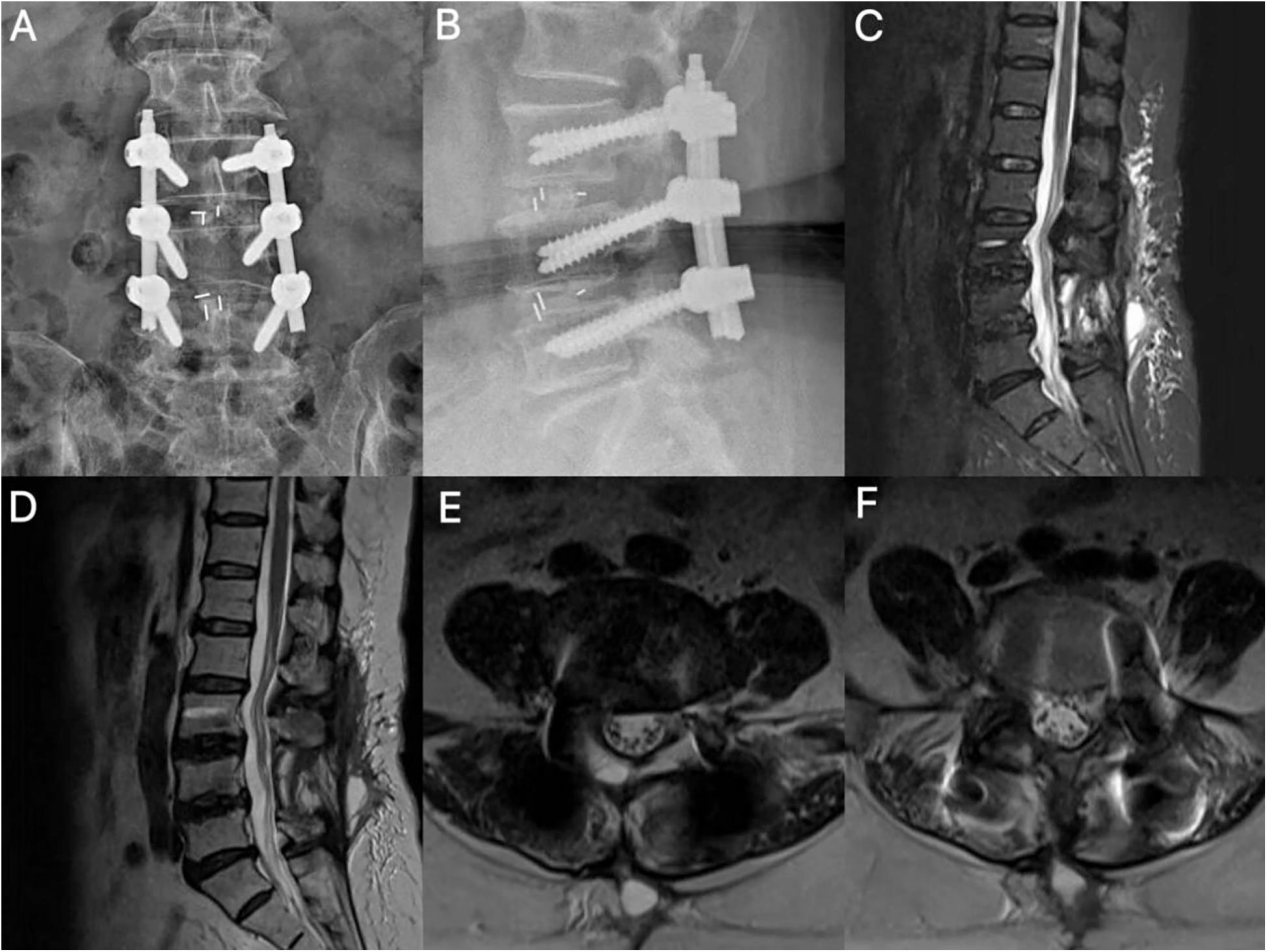
Postoperative imaging. **(A,B)** Lumbar radiographs demonstrate accurate positioning of L3–L5 pedicle screws and optimal interbody cage placement. **(C,D)** Sagittal MRI reveals spinal canal expansion and localized epidural fibrosis. **(E,F)** Segments L3–4 and L4–5 are shown with no space-occupying lesions; note the ventral cauda equina apposed to the dural sac in a hypertension state with a positive sedimentation sign (+).

On postoperative day 2, the patient reported significant relief of bilateral lower limb pain, with the VAS score improving to 3 points, representing a marked improvement compared to preoperative levels. By day 5, the VAS score worsened to 7 points, and new-onset bilateral lower limb numbness (particularly around the knees) accompanied by shooting pain emerged. These symptoms showed no significant improvement after treatment with corticosteroids and neurotropic agents. On day 7, numbness progressed with the development of saddle anesthesia, and bilateral lower limb muscle strength decreased to grade IV (Table 1). Repeat imaging demonstrated satisfactory reduction of spondylolisthesis with appropriate cage positioning and screw placement on lumbar radiographs. Lumbar MRI revealed no spinal stenosis or nerve root compression, showing instead expanded spinal canal volume without space-occupying lesions or foraminal obstruction. However, axial MRI sequences displayed positive nerve sedimentation signs, and the cauda equina exhibited a hyperintense, high-tension state (Figure 2).

**Table 1:** Timeline showing the events and the neurological findings.

Time	The neurological findings
Pre-operation	Bilateral lower limb pain with intermittent claudication
2 days after operation	Bilateral lower limb pain was significantly relieved
5 days after operation	Sensory numbness of both lower extremities with pain and normal muscle strength
7 days after operation	The numbness symptoms of the lower limbs progressed and the sensory disturbance in the sellar region occurred, and the muscle strength of both lower limbs decreased to grade IV

## Discussion and conclusions

Traditionally, postoperative neurological complications following spinal surgery have been attributed to direct mechanical compression (e.g., hematoma, fusion device displacement) or ischemic injury. However, the pathological core of ILNBD lies in the pathological increase in neural axial tension, which can independently cause disease even in the absence of obvious space-occupying compression (6, 7). In this case, the patient's symptoms improved early postoperatively, suggesting that the initial decompression was effective (8). However, on the fifth day, the patient suddenly developed progressive worsening of sensory-motor deficits in both lower limbs and numbness in the saddle region. Imaging studies ruled out common causes such as hematoma, shunt displacement, or residual compression, but axial MRI showed a positive nerve descent sign (9, 10). This finding strongly suggests that the cauda equina has lost its normal floating relaxation state and is in an axial traction state, consistent with the characteristic imaging findings of bowstring disease.

Upon reviewing the surgical procedure and relevant literature, we believe that the occurrence of iatrogenic lumbosacral neural arch string disease in this case was most likely due to technical errors in the highly controlled intervertebral fusion technique. Although the spinal cord and nerve roots possess a certain degree of elasticity, the surgeon relied on experience to select the size of the fusion device, neglecting the stepwise testing of the intervertebral spreader, which led to excessive expansion of the intervertebral space. This maneuver directly caused sustained static traction on the nerve roots and cauda equina at the nerve root exit point, resulting in progressively increasing axial tension and ultimately leading to neural conduction blockage (11–13). From a neurobiomechanical perspective, the pathological process is not merely simple mechanical traction. Although the spinal cord and nerve roots possess some elasticity, neural tissue has a critical threshold for tolerating traction injury. When axial tension applied to the cauda equina and nerve roots exceeds their physiological range of extension, this persistent static traction first compresses the microvascular system within the nerves, obstructing blood flow. This leads to ischemia and hypoxia of the nerve fascicle sheath, causing ischemic damage to the nerves. These injuries ultimately result in neurological dysfunction, triggering symptoms such as pain and sensory abnormalities (12, 14). The diagnosis of ILNBD is often insidious because it lacks specific clinical manifestations and is easily confused with common postoperative conditions such as nerve root compression, epidural fibrosis, or even early inflammatory reactions (3). Symptoms often appear several days or even later after surgery and progressively worsen. Conventional dehydration and hormone therapy often yield poor results, further complicating diagnosis. Imaging is key to differential diagnosis. The diagnostic turning point in this case was the axial MRI: although sagittal and coronal views showed enlarged spinal canal volume, no mass lesions, and unobstructed nerve root exit, the axial plane clearly captured the characteristic “positive sedimentation sign” (15, 16). The sedimentation sign (Lack of Nerve Root Sedimentation Sign) refers to the failure of the cauda equina nerve bundle to settle to the dorsal side of the dural sac under the influence of gravity in the supine axial MRI, instead tightly adhering to the ventral sac wall, strongly suggesting that the nerves are in a state of axial high tension and have lost their normal floating relaxation (17). This sign is of significant value in identifying nerve traction injuries without obvious compression. Therefore, for patients who develop unexplained, progressive neurological dysfunction postoperatively, it is essential to carefully analyze axial MRI sequences to identify features of cauda equina syndrome, such as high tension and the sedimentation sign, to avoid misdiagnosis or missed diagnosis (18).

The key lesson from this case is that while restoring intervertebral disc height is an important goal of fusion surgery, it should not be pursued blindly. Overemphasizing height restoration while ignoring individual differences and neural tolerance is the primary technical risk leading to ILNBD. We should use annular tension as an important reference for individualized fusion. After endplate preparation, the spreader should be incrementally increased from the minimum height until the annulus reaches appropriate tension—where the instrument can slide freely without elastic recoil. Additionally, a fusion device 1–2 mm smaller than the test value may be selected to compensate for postoperative subsidence and account for residual intervertebral soft tissue space. This case highlights that iatrogenic manipulation can independently trigger ILNBD. Spinal surgeons must thoroughly understand the pathophysiological mechanisms, clinical manifestations, and imaging characteristics of ILNBD and incorporate them into the important considerations for postoperative neurological dysfunction.

## Data Availability

The raw data supporting the conclusions of this article will be made available by the authors, without undue reservation.
